# Regional working in the East of England: using the UK National Standards for Public Involvement

**DOI:** 10.1186/s40900-018-0130-2

**Published:** 2018-12-06

**Authors:** Elspeth Mathie, Helena Wythe, Diane Munday, Graham Rhodes, Penny Vicary, Paul Millac, Julia Jones

**Affiliations:** 1 Centre for Research in Public Health and Community Care (CRIPACC), University of Hertfordshire, Hatfield, AL10 9AB UK; 2 Public Involvement in Research group (PIRg), CRIPACC, University of Hertfordshire, Hatfield, AL10 9AB UK; 3 Patient and Public Involvement (PPI), INsPIRE, Bedfordshire and Peterborough, Cambridge, UK; 4 Public and Patient Involvement in Research (PPIRes), Norfolk and Suffolk, UK

## Abstract

**Plain English summary:**

Involving patients and members of the public to help shape and carry out research is recommended in health research in the United Kingdom (UK). There are a number of regional networks of Patient and Public Involvement (PPI) groups, which support the collaboration between researchers, patients and public members. We are a group of researchers, patients and public members who came together via a PPI regional network in the East of England to collaborate on a research study about the extent of feedback from researchers to PPI contributors.

The aim of this paper is to use the recently developed UK National Standards for Public Involvement to structure our thinking about what worked well and what did not, within our recently completed study. We believe this paper is one of the first to use the National Standards to structure a retrospective reflection on PPI within a study.

Our findings showed that there are benefits of regional working, including easier access to public members and bringing together researchers, public members and those who run PPI groups for research collaboration. The main challenges included involvement of people before studies are funded and working across organisations with different payment processes.

The National Standards for Public Involvement has provided a useful framework to consider how best to involve patients and members of the public in research and could be a helpful structure to reflect on successes and challenges in individual projects and also regional, national or international comparisons of PPI in research.

**Abstract:**

**Background**

Regional networks of Patient and Public Involvement (PPI) organisations, including academic institutions, health and social care services, charities, patient and public groups and individuals, can play an important part in carrying out health research. In the UK, recommendations by the National Institute of Health Research (NIHR) encourage the use of regional, collaborative networks with shared resources and training.

**Methods**

The newly developed UK National Standards for Public Involvement were used as a framework for a retrospective reflection of PPI within a recently completed research study which focused on feedback from researchers to PPI contributors. PPI contributors, those running PPI groups (PPI leads) and researchers involved in the study have contributed to this reflection by completing evaluation forms throughout the research alongside notes of meetings and co-authors’ final reflections.

**Results**

Results revealed a number of successes where the regional network was particularly useful in bringing together PPI contributors, those who lead PPI groups and researchers. The regional network helped researchers to get in touch with patients and members of the public. Challenges included involving people before funding and bureaucratic and financial barriers when working across different organisations in the region. The importance of working together in flexible, informal ways was key and on-going support for the PPI contributors was vital for continued involvement, including emotional support not just monetary. The first four National Standards of inclusive opportunities, working together, support and learning and communications were particularly useful as means of structuring our reflections.

**Conclusions**

To our knowledge, this is one of the first research studies to use the UK National Standards for Public Involvement as a framework to identify what worked well and the challenges of PPI processes. It is suggested that as more reflective papers are published and the National Standards are more widely used in the UK, many lessons can be learnt and shared on how to improve our Patient and Public Involvement within research studies. Evaluations or reflections such as these can further enhance our understanding of PPI with implications for regional, national and international comparisons.

## Background

There is international recognition of the need to report on the volume and reach of evidence of Patient and Public Involvement (PPI) in health research [1]. Regional networks of organisations, groups and individuals have been recommended as an important asset for facilitating involvement of members of the public in shaping and designing health research [2]. The aims of these regional networks “*may include coordinated strategic planning, pooling of resources and expertise, shared learning, and support between those who carry out patient and public involvement role*s” ^(p.3)^ and the benefits include the creation of “*critical mass of expertise, knowledge, resources and relationships*” ^(p.12)^ [2]. Connectivity and regional working was one of the recommendations from a review of PPI carried out on behalf of the Department of Health in England in 2015 [3, 4] and has international implications.

There are many terms to describe members of the public involved in research (rather than as study participants) and we have chosen to use ‘PPI contributor’. The term ‘PPI lead’ describes those who facilitate, organise or run groups of PPI contributors. INVOLVE is the national advisory group funded by the National Institute for Health Research (NIHR) in England to “*support active public involvement in National Health Service* (*NHS*), *public health and social care research*” [5]. One region in England which benefits from an existing network of organisations interested in PPI in health research was set up and co-ordinated by the Research Design Service East of England in 2009 [6–8]. The East of England (EoE) PPI Regional Working Group brings together PPI contributors, PPI leads from the different PPI groups around the region (including the National Health Service and voluntary sector) and researchers from Universities. Senior Programme Officers from INVOLVE attend regional meetings to provide support and a national viewpoint. The group has regular face to face meetings, and shares PPI research and training activities across the region to provide region-wide opportunities and to reduce duplication.

The aim of this paper is to look at how the regional network supported a particular research study and describes and evaluates the methods and processes used to work together and identifies the challenges encountered. During the life-time of this research study, the National Standards for Public Involvement in the UK [9] were being developed so we thought it timely that we reflect on how regional working measured up against these standards to deliver our study. We have used each of the six Public Involvement Standards to structure our reflections [10]. The research study ‘Impact of PPI: Completing the Feedback Cycle’ focussed on feedback from researchers to PPI contributors [11]. It was the first time the regional network in the East of England had been involved together in a research study, which was funded by the Collaboration for Leadership in Applied Health Research and Care (CLAHRC) East of England. The research explored the important issue of whether PPI contributors receive feedback [12, 13], and whether their advice or contributions had been received, were useful and had been used. The findings of this study are reported elsewhere [11]. Of significance to the current paper, ten PPI contributors from across the East of England were involved in initiating the research idea, the design of documentation, data collection, data analysis and dissemination of the PPI Feedback study and it is these PPI processes which are a focus of this paper.

## Methods

The research study ‘Impact of PPI: Completing the Feedback Cycle’ was a mixed methods study, with a survey followed by interviews with PPI contributors, PPI leads and researchers. The methods of this study have been previously reported [11]. Of relevance to this paper, the study involved a regional collaboration between 10 PPI contributors, 11 PPI leads and 12 researchers (plus EM/HW) across the East of England region, who attended up to six face to face meetings. The focus of this paper is to describe our retrospective reflections on the PPI process of this study.

The methods of capturing our reflections included; 1) self-completed paper evaluation forms, which were filled in at the end of three of the six face to face meetings during the research by PPI contributors, PPI leads and researchers 2) e-mail correspondence, telephone and face to face conversations between researchers (EM/HW) and PPI contributors 3) minutes/notes of meetings 4) reflective discussion at two of the six meetings 5) final reflections from the co-authors. The evaluation forms were used as on-going reflection to improve practice, ways of working together and for planning future meetings. This paper gathers together views from all of the reflections at the end of the research study. It provides a case study of how the National Standards can be used as a framework for reporting PPI within a research study.

### Ethics

Ethical approval for the PPI Feedback Study was received from the Proportionate Review Subcommittee of the North West – Liverpool Central Research Ethics Committee (REC 16/NW/0245: IRAS 203158) in April 2016 and an amendment for the extension in March 2017. The study was initially funded for 1 year (2016–17) with an extension (2017–18).

### Findings

The UK National Standards for Public Involvement (2018) have been used to structure our reflections and assist us to identify the strengths and challenges of regional working in carrying out PPI within a regional research study about PPI Feedback. The six standards are listed below in Table 1, each of the six standards has further examples/indicators.

**Table 1:** National Standards for Public Involvement [9, 10]

Standard 1: Inclusive Opportunities	
We offer public involvement opportunities that are accessible and that reach people and groups according to research needs.	
Standard 2: Working Together	
We work together in a way that values all contributions, and that builds and sustains mutually respectful and productive relationships.	
Standard 3: Support & Learning	
We offer and promote support and learning that builds confidence and skills for public involvement in research.	
Standard 4: Communications	
We use plain language for timely, two way and targeted communications, as part of involvement plans and activities.	
Standard 5: Impact	
To drive improvement, we capture and share the difference that public involvement makes to research.	
Standard 6: Governance	
We involve the public in our governance and leadership so that our decisions promote and protect the public interest.	

Source: https://www.nihr.ac.uk/news-and-events/documents/Public_Involvement_Standards_M arch%202018_WEB.pdf

(accessed 4.6.18, reproduced with permission)

#### Standard 1: Inclusive opportunities

Standard 1 focusses on inclusive opportunities and indicator 1.1 states *‘we involve people affected by and interested in the research at the earliest stage’* [10]. The PPI Feedback research study included six PPI groups and PPI contributors from across the East of England regional area which covers over 19,000 km2 with rural areas and public transport limitations. This makes face to face meetings and travel difficult, especially for those individuals with mobility issues. In order to discuss the initial research ideas, a face to face meeting was attempted but proved too difficult to arrange, so a teleconference was arranged in 2015 (approximately a year before the study started). However, in the pre-development phase when there was no guarantee of funding, PPI contributors used personal phones, compounded by the fact that some teleconference call rates were more expensive than standard calls and not everyone was comfortable with skype or videoconferencing and so toll-free telephoning was not always an option. In general, the lack of pre-study funding had implications for early involvement at this stage of the research process as there was a lack of resources and mechanisms available to arrange meetings and reimburse PPI contributors.

There is an on-going challenge of how to involve PPI contributors before studies are funded which goes against recommendations of early involvement and offering ‘inclusive opportunities’. In relation to Standard/indicator 1.1 and 1.2 “*we identify and address barriers to taking up public involvement in research*”, we identified reimbursement of time and expenses as a barrier to involvement at this pre-study phase. The researcher (EM) has reflected on the difficulty of asking PPI contributors for comments and ideas but at the same time admitting that there was no budget, particularly as there was no guarantee that the research would be funded. The need to manage expectations around payments limited the amount of involvement that the researcher felt she could ask from the PPI contributors. One PPI contributor pointed out, it is not good practice to develop research about PPI if the people involved cannot be paid (and expenses refunded) and the researcher agreed. Similarly, writing papers at the end of a study when the funding is over. One consequence maybe that researchers will approach and rely on established groups and PPI contributors who they already know and have an on-going relationship. This works against the ‘reach and inclusivity’ with the Public Involvement indicator/example 1.4 “*a research project team advertises for new people to get involved, rather than approaching the same people each time*” [10].

The PPI Regional Working Group was a positive facilitator in helping this research get started and to progress, as relationships and networks were already established, existing meetings (funded from other sources) enabled people to get together and research ideas could be discussed. Pre- or inter-project funding for PPI groups is crucial to enable these costs to be met rather than relying on people’s good-will. The research was initially funded for a year and then received further funding for an extension, but this uncertainty (for example short term contract researchers) had implications for forward planning of the research and PPI. Economic constraints aside, this early contribution and involvement from start to finish of the project is reflected positively by one PPI contributor:

“*Having been part of the teleconference group that initiated the idea of this project in 2015, I have attended subsequent teleconferences, participated in seminars, been a member of the project steering committee and co-presented at conferences and dissemination meetings at all times. I was made to feel an equal partner...*” (PPI contributor)

Diversity and inclusion is often a challenge for research projects and the debate about representativeness is on-going [14]. Public Involvement Standard 1 on inclusive opportunities emphasises the importance of involving a range of people, including people new to research and PPI. The research idea originated from the PPI Regional Working Group where PPI members tended to have been involved in PPI for some time, so we also requested recruitment of PPI contributors who were newer to PPI. As the research was a regional collaboration, we aimed to have at least two PPI contributors from each of the six groups and tried to encourage a range of PPI experience and ages. Some of us had worked together before but many had not. The Public Involvement Standards (indicator 1.5) suggests offering choice and flexibility in opportunities for PPI in research and in this study the researchers outlined possible PPI activities at the beginning in an attempt to manage expectations of what level of involvement was possible and what resources (financial, staffing) were available. However, there is a careful balance between the budget available (allowance per meeting, number of PPI contributors) and individual skills, desires to be involved, health and other home/life circumstances. In addition, the different PPI groups and PPI leads were constrained in their amount of involvement by their own financial stability and administrative support. Overall, it was positive that the various collaborators found their way through the bureaucratic structures and we advise managing expectations by having honest discussions at the outset of any research study.

#### Standard 2: Working together

Once the research was funded, a number of face to face meetings were held throughout 2016–18 as part of PPI in the research activities (i.e. for data analysis and discussion of findings). These meetings were also used for on-going reflection and evaluation of the PPI process. Each meeting had a different purpose with PPI leads, PPI contributors and researchers (outside the research team) from the six PPI groups contributing as appropriate. Smaller research team meetings took place to analyse data, discuss progress and plan dissemination. One researcher (EM) was at all meetings, with the other researcher (HW) at 4 of the 6 meetings. The details of these meetings are provided in Table 2 and the PPI reflective method is listed in the last column.

**Table 2:** Main Face to Face Meetings of the PPI Feedback Research Activities

Date	Purpose of Meeting – Research	Attendees	PPI Reflections
Meeting 1: July 2016	1. Discussion of data collection and data analysis. 2. Designing a PPI feedback audit/process/form for PPI leads and PPI contributors to complete	8 PPI contributors 5 PPI leads	Paper evaluation form
Meeting 2: November 2016	1. Discussion of findings. 2. Designing a local feedback form (for each PPI organisation)	9 PPI contributors 4 PPI leads 9 researchers	Paper evaluation form
Meeting 3: December 2016	Data analysis meeting – interview transcripts	4 PPI contributors	Oral discussion
Meeting 4: April 2017	Planning meeting for 2nd year of project	6 PPI contributors 4 PPI leads	Minutes
Meeting 5: July 2017	Meeting to co-design feedback tool or guidance	6 PPI contributors 6 PPI leads 6 researchers, 1 Senior Public Involvement Advisor INVOLVE	Paper evaluation form
Meeting 6: January 2018	Address reviewers Comments on Paper [11]/Plan dissemination event	3 PPI contributors	Oral discussion
Dissemination Event: March 2018	Dissemination of findings to other PPI groups/PPI contributors. Co-presentation.	41 attendees PPI/Researcher/health professionals	Paper evaluation form

Given the geographical size of the East of England, it was important to find a convenient community building in the centre of the region which was accessible for all. It was decided following discussions with PPI contributors that meetings should start no earlier than 10.30 am and end by 3.00 pm, the time included lunch in a separate dining room (with time for informal discussion) and coffee breaks. The research study benefitted by bringing together PPI contributors attached to different PPI groups (from different locations), those who led PPI groups and researchers who had used the groups, having everyone in the same room and hearing each other’s point of view. However, we aimed to have roughly the same number of PPI contributors and researchers at specific meetings, which we largely achieved by inviting higher numbers of researchers. The difficulty in recruiting researchers was mainly due to research and work commitments.

“*The amount of informal time built into the various meetings was both enjoyable and valuable (both for this project and for future working together*)” (PPI contributor)

*“It was refreshing and educative to spend time and have discussion with PPI reps from other groups that have different structures and different ways of working. Some things were challenging as they took me out of my comfort zone and made me think” (PPI contributor)*

and another PPI contributor remarked;

*“Working with other PPI members of the project group was challenging but rewarding as we all came from different backgrounds and were able to view problems from different perspectives” (PPI contributor)*

A selection of quotes from the evaluation forms which were completed after some of the meetings emphasises the importance of working together. The following comments were made in answer to the question “what was particularly useful about the meeting?”;

*“coming together of different views, everyone was encouraged to contribute” (Anon)*

*“meeting other groups and sharing best practice, learning from others, meeting members of our group, (as I am new!)” (Researcher)*

The meetings were run as informally as possible, with short presentations allowing plenty of opportunities for whole group and table discussions (4–6 people). The facilitator (EM) was very aware of trying to enable everyone to contribute. All meetings included those from two or all three stakeholders group i.e. PPI contributors, PPI leads and researchers who had worked with a particular PPI group. Those from the same PPI group sometimes worked together (on a table) for example; to design locally relevant processes for their group and sometimes individuals worked in mixed groups to produce generic recommendations. Some of the attendees knew each other well and others had not worked directly together before. The benefits of this type of working is that the group produced something together, something they had ownership of and something they were then going to take back to their wider networks to implement.

The PPI Feedback research study provided the opportunity to try different techniques for working together. One meeting (Meeting 4: Co-design of PPI Feedback tool or guidance; see Table 2) was based on the World Café approach [15, 16]. Four tables were set up with a facilitator on each (each facilitator had been briefed beforehand), the tables had paper table cloths (so that attendees could write on them) and had bowls of fruit and sweets. Each of the four tables had a theme ‘How’, ‘What’, ‘When’ and ‘How’ of Feedback and each group of people moved around from table to table adding to the discussion of the previous group. The role of the table facilitator was to welcome people to the table, record the discussion and summarise. The whole session allows everyone to have a chance to discuss the four themes, with flexible timings dependent on how discussions were progressing. At the end, each table summarised their discussion to the whole group. Initially, this technique worked well, however, after the first round a couple of the PPI contributors said they did not want to move to the next table (people had settled themselves) and due to mobility issues it was not easy to move around the room. It was decided that it was easier if the four facilitators moved tables instead and carried their flip chart paper with them. This demonstrated the importance of flexibility and adapting the technique to suit the group. It is also an indication that facilitators may need training when bringing such groups together. This was an extremely productive meeting which led to the content, development and production of *Guidance for Researchers: PPI Feedback* [17] which is a resource for researchers to improve their feedback to PPI contributors. The Guidance is being used by PPI groups in their training in the region and has been nationally disseminated.

#### Standard 3: Support and learning

This section covers four areas under support and learning; attending meetings, payment, emotional support and learning about research.

##### Attending meetings

The importance of honest and open communication between researchers and PPI contributors is vital in order to provide relevant support (Standard Indicator 3.2). Our study identified a number of ways in which people could be enabled to attend meetings and to stay involved throughout a study. These included transport, catering and communication. Transport needs were met by researchers offering lifts to meetings and balancing the needs of PPI contributors (who might not function well in the early morning) with the needs of researchers (who were often required to arrive early to set up rooms); pre-booking open return train tickets and providing cash to pay taxis from the station so no-one is out of pocket or pre-ordering and paying for a taxis (for those not able to stand/travel at rush hours). Special catering needs could be met by, for example, providing bottled water if requested and buying extra gluten-free foods from supermarkets when catering does not offer a good choice. Relevant communication is essential to keep PPI contributors informed and involved and may require chasing finance departments to check if, and when, expenses have been paid; providing personal mobile phone numbers in case there is a problem; offering to have meetings at people’s houses and communicating by method of choice (paper with pre-paid stamped addressed envelopes, texting, phoning or emailing. One PPI contributor who travelled by public transport (bus and train) from the more rural parts of the East of England was offered overnight accommodation and reported how this helped her to be involved:

*“..being able to enjoy it (meeting) and not feeling too overwhelmed by the travelling! So very grateful for the overnight stay” (PPI contributor)*

As researchers, we reflect;

“*Try as we would to consider every eventuality with every individual, some things we hadn’t anticipated. Remaining flexible and thinking through every practical solution, and impractical ones, meant that we eventually found the answer and learnt for the future in the proce*ss” (Researcher)

##### Payment

A significant barrier to regional working is the varied processes and payment structures for PPI contributors across the different PPI groups and organisations. The challenges of payment for PPI contributors is not a new issue. The research team, which included PPI contributors, decided that it felt fair for all PPI contributors to be paid the same amount for their time, based on INVOLVE guidance, but not necessarily the same rate as set by their own host organisations. Setting up processes for any payment was not easy due to institutional bureaucracy. Initially the researchers (based at the host organisation) hoped to pay the PPI organisations so that they could organise paying their own PPI contributors, but, the organisations preferred PPI contributors to be paid directly. In addition, during the life-time of our research study, the host University went through a number of changes in terms of procedures in response to the ‘Right to Work’ checks required by employing institutions. Eventually, the PPI administrator and Human Resources colleagues agreed that PPI contributors do not necessarily fit into usual employee categories and came up with a solution where we were able to pay people. However, there was one meeting at the start of the second year of the study, where it looked doubtful whether we could pay individuals at all due to the new changes in procedures. This was particularly frustrating for everyone, and confusing for the PPI contributors who we had previously been able to pay. One PPI contributor did not attend this meeting as s/he could not be reimbursed for time. As researchers we reflect:

“*We strove for equality and thanks to the efforts of determined colleagues we were able to obtain a degree of flexibility to enable our PPI contributors to remain involved” (Researcher)*

##### Emotional support

In addition, to the support in terms of payments, of pertinence to one of our PPI contributors is the emotional support provided by the research team; Standard 3: Indicator/example 3.2 *"a research team recognises that an involved member of the public may need emotional support as part of being involved in research, and addresses this"*. The PPI contributor reflected;

“*...payment does not stop that individual from being maybe unwell, worrying about a family member, taking their time to travel all the way there and back, potentially on their own, going home on their own, or going back to find, you know, that maybe their child or their husband or their mother or their aunt, or whoever they’re taking care of, is unwell, and they don’t have to deal with all of that guilt. Does that £150, £20 or £25 stop that? I know professionals are in the same scenario, but we’re talking about completely different dynamics, their invite to that participation is different. The actual intellectual, emotional property to that person’s experience is being given freely, but there is a price every time, every time you go in and delve inside yourself to give, it is a gift, because not everyone can do it, not everyone wants to do it” (PPI contributor)*

As researchers, it is important to remember individual motivations, expectations and needs for becoming involved in research and this PPI contributor reflected on the importance of researchers having empathy.

##### Learning about research

Standard 3 on support and learning includes the indicators “*we develop, deliver and monitor learning opportunities in partnership, for all involved in research*” (Indicator 3.4) and “*we actively learn from others, we build on what we have learned and share our learnin*g” (Indicator 3.5). Our research went some way towards this but also could have done more, however, we also acknowledge that there is a balance between training PPI contributors in research techniques and encouraging the lay perspective to be expressed [18].

Some of the PPI contributors in this research were involved in data collection and analysis. One PPI contributor (co-researcher) undertook two of the telephone interviews from her home with the researcher in attendance for support. The co-researcher had previous interview experience with the support of the same researcher. The researcher and PPI contributor had had a conversation about their roles for these interviews, was it a waste of time for the researcher to sit in and what was her role (also relating to Standard 2: Indicator 2.3*"We ensure there is shared understanding of roles, responsibilities and expectations, which may evolve over time"*)?

“*I saw myself primarily as support for the technical equipment and listened to the conversation but did not take part…very occasionally [PPI contributor] turned to me for clarification but otherwise their interview was a two way conversation. The conversation felt more relaxed with the two PPI contributors discussing us ‘researchers’ revealing things they might not have done if I was carrying out the interview*” (Researcher)

**Fig. 1:**
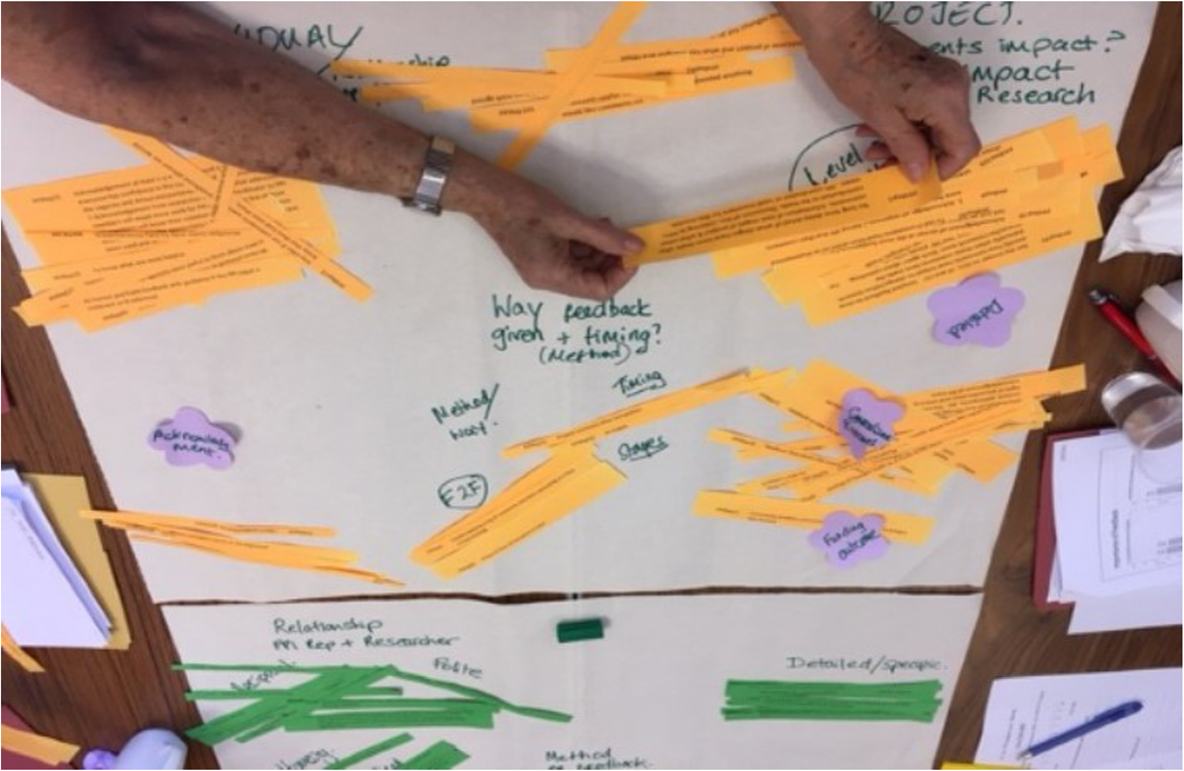
Carrying out manual data analysis

Collaborative working with PPI contributors and PPI leads who have mixed experience of qualitative data analysis was a challenge. Some needed support in understanding how to make sense of the data, some were able to identify what themes were important to them or what similar themes fitted together without needing to understand the whole process of qualitative analysis. We wanted to create ways for people to be involved who do not necessarily have or want separate research training. The PPI Feedback study aimed to involve all ‘voices’ together, it would not have been possible (physically or enough funding) to get this regional wide group together for a series of training days as well as the analysis. We used methods of qualitative analysis from the days before qualitative computer programmes, using paper and scissors to cut up printed interview transcripts or data excerpts from survey open responses and rearrange into similar themes by grouping the paper cuttings together. At one meeting the groups were given the cut up responses to the survey open question, “what is good feedback?”, each group discussed the different responses and physically placed similar themes together into piles (which were determined by the group), it was a form of open coding; (see Fig. 1)

*“Given the large amount of data this was an excellent and simple method for dealing with it – allowing everybody to easily participate”* (PPI contributor)

Two of the PPI contributors had not analysed interview transcripts before and they were given a couple of interview transcripts to read and asked to reflect on the main themes. The researchers took time to explain what to do, we wanted to know from their experience (or others’ experience) what were the interviewees trying to say, what were their main points, what did they feel was interesting or important in the interview? The themes identified by the researchers were either verified by the PPI contributors or further themes emerged;

*“I also studied and analysed interview responses, all of which were new to me and were enjoyable challenges helped by xx and xx [researchers], without whose help and enthusiasm I would not have achieved my personal improvement as a PPI representative*” (PPI contributor)

In terms of interview analysis reporting, one of the PPI contributors produced a word file which summarised the main themes as well as returning the paper interview transcripts with highlighted sections and references to their identified themes. Other members preferred to discuss the themes over the phone with a researcher making notes. In addition, a face to face meeting for PPI contributors and researchers was held where the themes and meanings were discussed in more detail.

#### Standard 4: Communication

The research focus on researchers providing feedback to PPI contributors created a heightened awareness amongst all those involved about providing appropriate and tailored communication. As the PPI Feedback research findings suggested, PPI contributors may want different types of feedback (a thank you, information on study success and progress and the impact of their involvement) [11]. The two researchers (EM/HW) tried to acknowledge all correspondence, provided on-going updates of study success (funding, ethics), study progress through newsletters, updated website and also let individual members know what impact their contribution had had on the research by sharing notes of minutes, track changes on documents and final versions of documents. It is also acknowledged that the researchers were not always clear in their communication and care must be taken in wording emails and taking the time to discuss issues on the phone.

The advantage of being part of the regional network, meant that the wider research dissemination and feedback to the collaborating groups took place through a number of face to face, physical and electronic routes. PPI leads were able keep their local groups up to date with progress. The PPI Regional Working Group was kept informed by short presentations throughout the study and the lead researcher (EM) also presented the research to individual PPI groups at the beginning and end of the study where possible. Newsletters were emailed or posted out via the PPI leads. The PPI Regional Working Group, with access to existing databases (via the PPI leads), meant that communication around the region was accessible and assisted the distribution of surveys and recruitment of participants whilst maintaining anonymity. The PPI contributors encouraged wider communication in terms of dissemination, and as researchers we felt it important not only to write in academic journals but also newsletters and blogs. It is suggested that others undertaking similar regional work may find existing networks useful as a starting point for collaborative working, as well as developing new ones over time.

#### Standard 5: Impact

There have been numerous ways PPI contributors have had an impact on this research, from suggesting the focus of the study, to shaping survey and interview questions and data analysis. It is recognised that sometimes it is difficult to identify PPI impact, especially in meetings where everyone contributes. However, attempting to document impact as the study progresses is key. In the Feedback Cycle study, PPI contributors made numerous changes to documents, helped to draft survey questions; for example, the term “on-going dialogue” was added as an option for feedback in the list of survey responses. PPI contributors made suggestions and directed the emphasis for data collection.

“*One PPI contributor felt that feedback from researchers was needed less as time progressed, another PPI contributor felt it was always necessarily, it was decided to explore this theme in more depth in the interviews*” (Researcher)

On reflection, one researcher stated;

“*There is always more we can do, wanting to involve people more and more meaningfully, but there are lots of trade-offs, time is often so tight (wanting to give PPI contributors long enough to read documents but also having a good enough draft before a deadline), reciprocal relationships are key*” (Researcher)

The PPI contributors had different skills to bring to the research process and ensured the research focussed on what was important to them. They also encouraged the researchers to ‘be bold’ and continue to develop and promote the research, not only within the region but nationally. It is important to value the skills and perspectives of all, and future studies could report the ‘skills mix’ of all those involved.

#### Standard 6: Governance

Standard 6 on governance includes the indicator *6.1 "Public voices are heard, valued and included in decision making"*. In our PPI Feedback study, PPI contributors were involved in a number of key decisions from the start, from deciding the research topic to the content and format of conference presentations. Indeed there was involvement at every stage of the research process and it is fair to say the PPI contributors influenced the direction and focus of the study from the outset. On reflection as a team we welcomed and appreciated the opportunity to make, validate or change decisions, for example;

“*...we decided together not to include a definition of ‘feedback’ in the survey and interviews and let participants provide their own definit*ions” (Researcher)

“*I felt there was a strong commitment to power sharing alongside continuous effort to put it into practice from the researchers throughout*” (PPI contributor)

“I always referred to *our or your* study rather than *my study*” (Researcher)

We attempted to document decision-making within our study and it is suggested there should be further exploration of decision-making in future studies, where and by whom decisions are made and which decisions are made by consensus.

One example was our presentation at the INVOLVE conference (2017) which took the form of a playlet which was written by one of co-researchers (GR) and presented by three individuals (PPI contributor, PPI lead and researcher (EM)). As we were allocated the session after lunch and near the Christmas period, GR felt we needed something to wake people up and not rely on PowerPoint, so he suggested a pantomime theme. The three presenters met a couple of weeks before the conference to have a run through and also met on the day for a further practice.

“*I felt completely out of my comfort zone, acting rather than presenting purely by PowerPoint, but it was completely appropriate, the playlet clearly explained the rationale for the project, made the session fun and memorable and I am very glad we went with GR’s suggestion, GR judged the mood of the conference and audience well*” (Researcher)

“*...my innovative ideas were embraced e.g. authors of playlet at two conferences*” (PPI contributor)

Here is an overall reflection from one of the researchers on using the National Standards for Public Involvement;

“*Reflecting on the National PPI standards has been a helpful exercise in identifying the barriers and facilitators to implementation during this study. We have fallen short of meeting some standards as the reality of PPI is often more muddy, complex and nuanced in relation to people’s lives and institutional processes. The standards are however what we should aspire to in thought and intention and when practicalities and logistics fail us, communication and explanation is key*” (Researcher)

## Discussion

Our research took place during the period of consultation about the National Standards for Public Involvement in the UK which were launched in March 2018 [9] and are still being tested in pilot sites and debated [19]. To our knowledge, we are one of the first research teams to use the National Standards for Public Involvement as a framework to evaluate PPI in research including different perspectives – PPI contributors and researchers. Our work together, our thinking and reflections, framed within our interpretion of these Standards, has helped to highlight which areas went well, those for improvement and potential gaps, especially focussing on the process issues of PPI within a research study. It has been a retrospective exercise but will also be extremely useful for planning future studies.

There are many areas of the UK which are now benefiting from regional working in terms of Patient and Public Involvement. Sharing examples of regional working is useful for learning what works well, such as in the West of England [20] and 'share-bank' in the Midlands [21]. Our reflections demonstrate many advantages of regional working; firstly being able to access existing PPI groups and individuals to discuss early research ideas; secondly, the PPI leads can help identify potential PPI contributors, distribute research tools (such as surveys), support the dissemination of findings and linking in with local organisations and services; and thirdly, provide peer support and information sharing for PPI contributors, PPI leads and researchers.

The first four National Standards - inclusive opportunities, working together, support and learning and communications - were considered particularly useful for examining process issues of PPI within a research study although we found some overlap between some of the Standards. It was beneficial to have a clear ‘research task’ for furthering activity and continued the working relationships and development of the PPI Regional Working Group. The research brought together the interested parties (PPI contributors, PPI leads and researchers) from the different areas of the region in the same room for a common purpose.

The many benefits of regional working reported in this paper were tempered by the logistical challenges in working across multiple organisations and their varying processes especially around payments to PPI contributors. More needs to be done at a higher level and within institutions, such as Universities and the National Health Service, to enable straightforward processes for paying and funding PPI work, especially pre-funding. Mockford et al. [22] reported similar bureaucratic difficulties involving co-researchers in a study on dementia. It is recognised that there are some grants or small amounts of money available for pre-study funding for PPI, however, there remain many challenges to involving a range of people at this pre-funding stage.

Whilst some studies have successfully trained PPI contributors in research skills and complex data analysis [23, 24], engaging members of the public in research has much to learn from participatory methods and engagement work. Researchers can support members of the public to take part in research methods and processes by using techniques which facilitate group work, such as the World Café which worked well for our research. Our research provided the opportunity to try out various group work techniques, to enable those with a range of research experience to work together, such as physically sorting cuttings of quotes. The need for flexibility was emphasised and recognising that PPI contributors often have to ‘dip in and out’ over the course of research for a variety of reasons. PPI contributors and researchers working together and hearing each other’s view point was particularly successful, and as a way of working has been used for some years by Macmillan Cancer [25].

As a way of reflecting and reporting on PPI within studies, researchers have started to use various methods or frameworks such as Giebel et al. [26] used the INVOLVE values [27], Jinks et al. [28] used the six salient actions from the RAPPORT study [13, 29, 30] and the GRIPP2 checklist [31]. The recent publication of the INVOLVE co-production guidance [32] also made us reflect on whether the research was co-produced and although many elements could be termed co-production, with many decisions being shared, ideas co-produced, equal and reciprocal, the lead researcher (EM), who had responsibility for the funding, recognises that the research was not fully ‘co-produced’ [33] with some decisions not jointly owned. There were also elements of the research where decisions were made and the end result was not necessarily attributable to a PPI contributor or a researcher but a combination of shared discussion, such as the development of the Guidance for Researchers: PPI Feedback [17] .

## Conclusion

This paper has illustrated how the National Standards for Public Involvement in the UK can provide a useful framework for reflecting on PPI within a research study. The framework provided a structure to identify areas that worked well and also reflect on where there is room for improvement. Using a standardised framework can help individual research studies to reflect on the PPI process and also allow comparison between research studies and organisations at the regional, national and international level. There is much potential for improving our PPI activity through collaboration, with the growing number of regional networks and relationships between PPI organisations, Universities, voluntary sector and health and social services, whilst acknowledging the need to improve the bureaucratic and financial structures that continue to hinder PPI in ‘real-world’ research. PPI contributors, PPI leads and researchers need to continue to work and learn together, whilst also recognising and reporting the challenges [34].
